# The Multiple Object Test as a performance-based tool to assess the decline of ADL function in Parkinson’s disease

**DOI:** 10.1371/journal.pone.0200990

**Published:** 2018-08-01

**Authors:** Aline Beyle, Hannah Glonnegger, Bernhard Cerff, Susanne Gräber, Daniela Berg, Inga Liepelt-Scarfone

**Affiliations:** 1 German Center of Neurodegenerative Diseases (DZNE), Tübingen, Germany; 2 Hertie Institute for Clinical Brain Research, Department of Neurodegeneration, University of Tuebingen, Tübingen, Germany; 3 Department of Neurology, University Hospital Bonn, Bonn, Germany; 4 Department of Neurology, Christian-Albrechts-University, Kiel, Germany; Nathan S Kline Institute, UNITED STATES

## Abstract

**Introduction:**

As cognitive-driven worsening of activities of the daily living (ADL) in Parkinson’s disease (PD) is the core feature of PD dementia (PDD), there is great need for sensitive quantitative assessment. Aim of our study was the evaluation of cognitive-driven worsening of ADL by the performance-based Multiple Object Test (MOT), offering an essential clinical advantage as it is quick and easy to apply in a clinical context even on severely impaired patients.

**Methods:**

73 PD patients were assessed longitudinally over a period of 37 (6–49) months. According to their neuropsychological profile the sample was divided into two groups: PD patients with (n = 34, PD-CI) and without cognitive impairment (n = 39, PD-noCI). The MOT comprises five routine tasks (e.g. to make coffee) quick and easy to apply. Quantitative (total error number, processing time) and qualitative parameters (error type) were analyzed using non-parametric test statistic (e.g.Wilcoxon signed-rank test, binary logistic regression).

**Results:**

Median number of total errors (p = 0.001), processing time (p<0.001), perplexity (p = 0.035), and omission errors (p<0.001) increased significantly from baseline to follow-up in the total sample. Worsening of MOT performance was correlated to cognitive decline in the attention/ executive function and visuo-constructive domain. PD-CI showed an increase in omission errors (p = 0.027) compared to PD-noCI over time. This increase in omission errors between visits was further identified as a risk marker for PDD conversion.

**Conclusion:**

The MOT, especially frequency of omission errors, is a promising tool to rate PD patients objectively and might help to identify patients with a high risk for having mild cognitive impairment or dementia.

## Introduction

Impairment in activity of daily living (ADL) function is the most crucial characteristic to differentiate between mild cognitive impairment and early stage dementia [1, 2]. In Parkinson’s disease (PD), both motor and cognitive impairment have the potential to affect patients ADL function [3, 4]. ADL impairment indicative for Parkinson’s disease dementia (PDD) is required to be primarily related to cognitive but not to motor skills [1]. Minor ADL impairment can also be observed in PD patients with mild cognitive impairment (PD-MCI) [5–7], which normally does not interfere with patients`daily life, suggesting that both ADL and cognitive function are present to a mild extent in the prodromal phase of PDD. Especially more complex instrumental ADL (using the telephone, managing finances, and medication etc.) can be impaired in early stages of cognitive impairment, whereas basic ADL can be preserved for a long time in the course of PDD [5].

It is often difficult for PD patients and their caregivers to understand the primary reason for ADL impairment. As PD is primarily a movement disorder, the influence of cognitive deterioration on ADL impairment might not always be obvious for raters. It is therefore difficult for the physician to gather valid information about cognitive-driven ADL impairment, especially in early stages of PDD. Moreover, PD patients might not always be fully aware of their deficits [8, 9]. These major limitations affect the validity of ADL questionnaires, often used in the clinical daily routine. Therefore, objective tests -economic in time and cost- with a high diagnostic accuracy are needed.

Performance-based tests are a promising tool to discriminate between different levels of cognitive impairment in PD [6, 10, 11]. So far, only a few studies have focused on the evaluation of performance-based ADL tests for the early and valid diagnosis of PDD [7, 12]. Recently, we confirmed good diagnostic accuracy of the Multiple Object Test (MOT), a quick and easy to apply performance-based ADL test for PDD [13]. Besides quantitative aspects, the MOT offers the possibility to evaluate qualitative characteristics of ADL dysfunction, e.g. the type of error committed like the Naturalistic Action Test [12].

It has been speculated that the MOT has the potential to predict progressive cognitive decline [13]. Thus, aim of the study was to evaluate changes in the MOT over 3 years in a group of PD patients with and without cognitive impairment. We hypothesized that PD patients with cognitive impairment commit more errors in the MOT at both, baseline and follow-up assessment and are more prone to faster deterioration in the MOT, associated with worsening cognitive function over time.

## Methods

### Participants and recruitment

In total, 131 idiopathic PD patients diagnosed according to the United Kingdom Parkinson’s Disease Society Brain Bank criteria [14] were recruited from the outpatient Parkinson’s clinic of the University hospital of Tuebingen. Further inclusion criteria were age ≥46 years, German as mother tongue, absence/ correction of visual or hearing impairment, no other diagnosed diseases of the central nervous system, a stable health status that allows for comprehensive testing and a Mini-Mental State examination score ≥18 to ensure capacity to consent. Patients with history of alcohol or drug abuse and deep brain stimulation were excluded from study participation. The study was approved by the ethics committee of the Medical Faculty, University of Tuebingen, Germany; all patients gave written informed consent.

### Assessments

All assessments were performed twice–at baseline and after a median of follow-up interval of 37 (6–49) months. Assessments were performed within one session (˞ 4 hours duration). If patients were too disabled, testing was conducted in their respective home. Motor function was assessed by a neurologist. Neuropsychological assessment was conducted by a neuropsychologist or trained research staff.

#### Clinical data, motor function, and depression

Demographics (age, sex, years of education), medical history (duration of Parkinson’s disease), and current medication status expressed as levodopa equivalent daily dose [15] were registered. To assess motor function, the Unified Parkinson’s Disease Rating Scale part III (UPDRS-III) [16] and the modified Hoehn & Yahr scale (H&Y) [17] were applied. The Beck Depression Inventory I (BDI) was used to screen for signs of depression [18].

#### Neuropsychological test battery

Two global cognitive screening scales were performed: the Mini-Mental State Examination (MMSE) as part of the completely executed Consortium to establish a Registry for Alzheimer’s Disease (CERAD) plus battery [19] and the Parkinson Neuropsychometric Dementia Assessment (PANDA) [20].

A comprehensive battery was applied to test for cognitive function. *Executive function*: Trail Making Test (TMT) part B [19], Digit Span Forward of the Wechsler Memory Scale-Revised Edition (WMS-R) [21], and the Figure Test of the Nuernberger-Alters-Inventar (NAI) [22]; *Attention*: Digit Span Backward of the WMS-R [21] and the TMT part A [19]; *Memory*: Word List Memory, Word List Recall, Word List Intrusion, and Discriminability of the CERAD; *Visuo-construction*: Praxis and Praxis Recall of the CERAD, and Object Decision of the Visual Object and Space Perception Battery (VOSP) [23]; *Psychomotor speed and language ability*: Verbal Fluency Test and the Boston Naming Test of the CERAD.

#### Assessment of ADL functions

Impairment of ADL functions was evaluated using the MOT as described by De Renzi and Lucchelli [24]. Only one task was slightly modified; instead of preparing an espresso, subjects were asked to prepare a cup of coffee.

The MOT consists of five different routine tasks: (1) lighting a candle, (2) opening a padlock, (3) drinking a glass of water, (4) preparing a letter ready for mailing and (5) preparing a cup of coffee. Patients were equipped with the objects needed to fulfill the corresponding task (e.g. padlock and key) and were verbally instructed by the examiner of the required task. Subjects were videotaped for MOT analysis. MOT ratings were evaluated blinded to the clinical and neuropsychological assessments. Inter-rater Reliability (IRR) was assessed for 87 participants, of whom ratings of a second independent rater were available.

Rating included total number of errors with a maximum score of 25, as well as total processing time, which is the sum of length of time to complete each single task, as quantitative parameters and different error types as qualitative parameters. We distinguished the following error types: perplexity (disorientation and confusion how to accomplish the task, trial and error actions), omission (a specific element of action was left out), mislocation (correct usage of an object in an inappropriate location), misuse (incorrect usage of an object), sequence error (element of action is performed at the wrong time in the action process), and clumsiness (dexterity errors). Clumsiness errors were counted but not further analyzed, since this error type is easily biased in PD due to motor impairment. The total number of errors was calculated by summing up all errors made by every PD patient (one error per task with a maximum of five for each error type per patient).

### Classification of cognitive groups

Diagnosis of the two groups according to their cognitive status was based on the guidelines of the Movement Disorder Society Task Force [1, 2]. PDD was classified as follows: cognitive impairment in at least one neuropsychological test in two domains (test result <1.5 SD of norm population) and the clinically rated impact of ADL function. Patients were diagnosed with PD-MCI, if their performance was impaired in at least two tests (test result <1.5 SD of norm population) of the neuropsychological test battery (see S1 Table) with impairment not being potent enough to interfere severely with ADL function. A neuropsychologist and a physician performed the clinical ADL rating in a personalized interview with patients and caregivers. Patients who did not meet criteria for PD-MCI or PDD were classified as PD patients without mild cognitive impairment (PD-noCI). Furthermore, PD-MCI and PDD were combined as PD patients with cognitive impairment (PD-CI).

### Statistical analysis

Data were analyzed using IBM SPSS Statistics 22.0 for Windows. Descriptive data are given as number (percentage) or median (range). Between-group comparison of demographic and clinical data was performed by non-parametric test statistic (Mann-Whitney-U-Test and Wilcoxon signed-rank Test (2-sided)).

Inter-rater reliability and correlation analysis were based on the Spearman-rank correlation coefficient (rho). Correlation analysis was performed comparing calculated change scores (baseline–follow-up values) of the UPDRS and neuropsychological data with change score of the MOT. In the total sample, the Wilcoxon signed-rank test (2-sided) was applied to compare baseline and follow-up median performance of quantitative (total processing time, number of errors) and qualitative MOT parameters (number of specific errors committed).

To compare MOT performance between PD-noCI and PD-CI, logistic regression models were performed with classification of cognitive group as dependent and MOT parameters as well as the following covariates as independent variables: years of disease duration, UPDRS-III, and BDI. Three separate models were analyzed for baseline, follow-up, and the calculated change score between visits, including either i.) total number of errors, ii.) total processing time or iii.) all qualitative MOT parameters as independent variables.

## Results

The median age of the whole patient group at baseline was 70.6 (46–89) years; 113 (86.3%) were male (see S2 Table for details). Of the 131 participants, 73 were assessed within a follow-up interval of 37 (6–49) months. Reasons for study drop-out were: unstable health status (n = 17, 13.0%), refusal to participate at follow-up (n = 21, 16.0%), death (n = 10, 7.6%), deep brain stimulation after baseline visit (n = 5, 3.8%), and failure to re-contact due to unknown address (n = 5, 3.8%). Patients lost to follow-up were older (*P* < .001), scored higher at the BDI (*P* = .026) and suffered from more severe motor problems documented by both UPDRS-III (*P* = .004) and H&Y stage score (*P* < .001).

### Clinical characteristics of the follow-up cohort

At baseline, 26 (35.6%) of all 73 patients were diagnosed with PD-MCI, 8 (11%) with PDD and 39 (53.4%) had no cognitive impairment (PD-noCI). At follow-up, 8 (11%) were newly diagnosed as having PD-MCI and 8 (11%) as PDD (see Table 1 for details). Five (6.8%) patients with baseline diagnosis of PD-MCI reversed to PD-noCI at follow-up, resulting in a total number of 21 (28.8%) patients classified as PD-MCI, 16 (21.9%) as PDD, and 36 (49.3%) as PD-noCI with follow-up data.

**Table 1:** Cognitive diagnosis of patients at baseline and follow-up.

		Follow-up		
		PD-noCI	PD-MCI	PDD
Baseline	PD-noCI	PD-noCI stable	PD-MCI new-onset	
		31/42.5%	8/11%	
	PD-MCI	PD-MCI reversible	PD-MCI stable	PDD new-onset
		5/6.8%	13/17.8%	8/11%
	PDD			PDD stable
				8/11%

Values are given as number and relative frequency. PD-noCI, PD patients without cognitive impairment; PD-MCI, PD patients with mild cognitive impairment; PDD, PD dementia

Due to the low number of PDD patients at baseline in our sample, PD-MCI and PDD patients were summarized as PD-CI for further analysis. Details of baseline demographic and clinical data are presented in Table 2. In summary, patients classified as PD-CI had a longer disease duration (*P* = 0.037) and more severe motor problems reflected by higher UPDRS-III scores (*P* = 0.004) than PD-noCI. BDI scores were not significant (P = 0.07), however, since significance was borderline and depression is known to have an impact on cognition and ADL [25] it was included as possible confounding covariate.

**Table 2:** Baseline characteristics of the follow-up cohort, and between-group comparison of Parkinson’s disease patients with (PD-CI) and without (PD-noCI) cognitive impairment.

	Follow-up	PD-noCI	PD-CI	*P* value*
Number, n/%	73/100	39/53	34/47	
Age in years	69.3/46-79	67.9/51-77	70/46-79	.13
Male gender, n/%	66/90.4	35/89.7	31/91.2	.84
Years of education	12/6-20	13/8-19	11.8/6-20	.11
Years of disease duration	6/1-22	5/1-18	7.5/2-22	**.037**
LEDD	560/100-2743	560/100-1320	560/100-2743	.95
UPDRS-III	22/7-55	20/7-49	29/10-55	**.004**
Hoehn and Yahr stage, n/%				.07
1/1.5	12/16.4	9/23.1	3/8.8	
2/2.5	49/67.1	27/69.2	22/64.7	
3	9/12.3	3/7.7	6/17.6	
4	3/4.1	0/0	3/8.8	
BDI	8/0-34	6/0-24	9/1-34	.07

If not other indicated, values are given as median/ range. n, Number; %, percentage; LEDD, Levodopa equivalent daily dose; UPDRS, Unified Parkinson’s Disease Rating Scale; BDI, Beck Depression Inventory.

*Significant *P*values (*P* < .005) are given in bold.

Neuropsychological data are reported in S1 Table. As expected, PD-CI patients showed more cognitive impairment than PD-noCI, indicated by lower test scores in almost all neuropsychological tasks (*P*<0.05). Exceptions were the Digit Span Forward, subtest of the WMS-R, the two Praxis subtests of the CERAD, and Verbal Fluency, for which no statistically significant group differences between PD-noCI and PD-CI were detected.

### Inter-rater reliability of the MOT

IRR based on a subset of 87 PD patients (PD-noCI: n = 41, 47.1%, PD-MCI: n = 35, 40.2% and PDD: n = 11, 12.7%). IRR of total MOT processing time (rho = 0.97) and total number of errors (rho = 0.77) was high. Among the qualitative parameters, IRR was highest for total number of omission (rho = 0.78) followed by misuse (rho = 55), mislocation (rho = 0.50), perplexity (rho = 0.49), and sequence errors (rho = 0.26).

### Baseline and follow-up MOT performance in the total follow-up cohort

In all PD patients with follow-up assessment, all quantitative parameters, that is total number of errors (*P* = 0.001) and total processing time (*P*<0.001) were significantly higher at follow-up (see Table 3 for details). The median number of perplexity (*P* = 0.035) and omission errors (*P*<0.001) increased significantly from baseline to follow-up assessment (Table 3).

**Table 3:** Parameters of the Multiple Object Test of follow-up cohort.

	Baseline	Follow-up	*P*value*
Quantitative parameters:			
total error number	2/0-13	3/0-13	**.001**
total processing time	164/95-450	204/88-694	**< .001**
Qualitative parameters:			
perplexity errors	0/0-3	1/0-4	**.035**
omission errors	0/0-4	1/0-4	**< .001**
mislocation errors	0/0-2	0/0-3	.32
misuse errors	0/0-4	0/0-2	.49
sequence errors	0/0-2	0/0-2	.15

Values are given as median/ range.

*Significant *P*values (*P* < .005) are given in bold.

At baseline, the most frequent errors were perplexity (34.4%), omission (28.6%), and sequence errors (17.5%). Omission errors (32.8%) were the most frequent errors at follow-up, followed by perplexity (29.2%), and sequence errors (22.7%).

### Change in MOT performance among cognitive groups over time

Our logistic regression model (Table 4) showed that at both examinations, the total number of errors (*P* = .016/*P* = .002) and total processing time (*P* = .006/*P* = .014) were higher in PD-CI than in PD-noCI. The logistic regression models including qualitative parameters to predict group membership, revealed that PD-CI patients had more perplexity (*P* = .008) and mislocation errors (*P* = .048) compared to PD-noCI at baseline. At follow-up only the number of perplexity errors (*P* = .008) differentiated significantly between PD-noCI and PD-CI.

**Table 4:** Comparison of MOT parameters of PD patients with (PD-CI) and without (PD-noCI) cognitive impairment at baseline and follow-up visit.

	Baseline			Follow-up		
	PD-noCI	PD-CI	*P*value*	PD-noCI	PD-CI	*P*value*
Number, n/%	39/53.4	34/46.6		39/53.4	34/46.6	
Quantitative parameters:						
total error number	1/0-5	3/0-13	**.016**	2/0-6	4/0-13	**.002**
total processing time	151/95-226	201.5/105-450	**.006**	177/115-501	292/88-694	**.014**
Qualitative parameters:						
perplexity errors	0/0-1	1/0-3	**.008**	0/0-2	1/0-4	**.008**
omission errors	0/0-3	0.5/0-4	.63	0/0-3	1/0-4	.23
mislocation errors	0/0-2	0/0-2	**.048**	0/0-1	0/0-3	.15
misuse errors	0/0-1	0/0-4	.53	0/0-1	0/0-2	.92
sequence errors	0/0-2	0/0-2	.67	1/0-2	0/0-2	.05

If not other indicated, values are given as median/ range. MOT, Multiple Object Test; n, Number; %, percentage; PD-noCI, PD patients without cognitive impairment; PD-CI, PD patients with cognitive impairment.

*Significant *P*values (*P* < .005) are given in bold.

**Fig 1:**
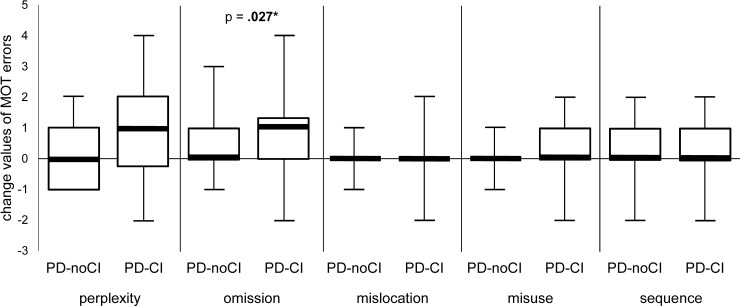
Progression of qualitative MOT parameters. Comparison of change values (follow-up–baseline) in each Multiple Object Test (MOT) error category between PD patients with (PD-CI) and without (PD-noCI) cognitive impairment.

According to the change scores, neither increase in total error number (*P* = .08) nor total processing time (*P* = .07) statistically differed between PD-noCI and PD-CI. Progression of qualitative MOT parameters among the cognitive groups from baseline to follow-up is shown in Fig 1. PD-CI showed a significant increase in omission errors (*P* = .027) compared to PD-noCI over time.

### Post-Hoc analysis: Change in MOT performance over time as a progression marker

To evaluate whether omission errors are associated with new onset PD-MCI (n = 8, 20.5%) among PD-noCI and/ or new onset PDD (n = 8, 12.3%) at follow-up, four logistic regression models were performed with either baseline status or increase in omission errors, and the following covariates as independent variables: years of disease duration, UPDRS-III and BDI.

Baseline omission errors did not predict conversion to PD-MCI and PDD (*P*>0.05 respectively with only 21% and 17% variance explained by the model). However, an increase in omission errors from baseline to follow-up was associated with new onset PDD at follow-up (*P* = .011, 43% variance explained), but not with progression from PD-noCI to PD-MCI (*P* = .68, 25% variance explained).

### Correlation analysis between clinical markers and MOT performance

To evaluate the relationship between change in the MOT and in the cognitive and motor performance change scores between baseline and follow-up assessment were correlated between parameters (see S3 Table). Correlation analysis between MOT and UPDRS-III change scores only showed a marginal association between increase of the UPDRS-III score and increase of total processing time (rho = 0.23, *P* = .05) as well as misuse errors (rho = 0.23, *P* = .05) between the two examinations.

Correlation analysis revealed a significant association between change of MOT parameters and change of neuropsychological tests targeting attention, executive function, visuo-construction, psychomotor speed and naming performance: Total error number and Object Decision (rho = -0.28, *P* = .018); processing time and TMT part A (rho = -0.36, *P* = .002) as well as Praxis Recall (rho = -0.26, *P* = .024); perplexity errors and Digit Span Forward (rho = -0.27, *P* = .020); omission errors and Boston Naming Test (rho = 0.28, *P* = .018); mislocation errors and Verbal Fluency (rho = 0.30, *P* = .011) as well as Boston Naming Test (rho = 0.28, *P* = .017); misuse errors and Praxis Recall (rho = -0.25, *P* = .030); sequence errors correlated significantly with Praxis Recall (rho = 0.28, *P* = .018) and worsening of the Object Decision task (rho = -0.30, *P* = .011).

## Discussion

Aim of the study was to evaluate worsening of cognitive-driven ADLs by using the performance-based MOT test. To the best of our knowledge, this is the first longitudinal study using performance-based instrumental ADL testing in PD.

Our results revealed a high IRR (>0.75) for total processing time and number of errors, confirming that the MOT is a reliable assessment for ADL impairment in PD. Among the five different error categories of the MOT, omission errors showed highest IRR.

These most reliable parameters (total processing time, total errors, and omission errors) in addition to perplexity errors showed a significant increase in our PD sample at follow up within three years, supporting previous reports that ADL function declines over the whole course of PD [5, 26]. We therefore conclude that the MOT is a useful tool for measuring progression of ADL impairment in PD patients over time.

Decline in ADL function has been associated with both, motor and cognitive impairment in PD [3, 4]. Especially, decline in instrumental ADL function in PD has been previously stated as a risk marker for cognitive impairment and dementia [27]. Our correlation analysis claims that change in MOT parameters primarily reflect cognitive decline. Progression in quantitative MOT parameters reflected worsening of attention and visuo-construction. Perplexity errors were associated with executive dysfunction. Omission and mislocation errors conveyed an association with language and psychomotor speed. Change in misuse und sequence errors were closely linked to visuo-constructive cognitive performance. This provides support for previous reports on a strong relationship between memory, executive functioning, processing speed and instrumental ADL function [28] as well as for longitudinal studies proposing these cognitive domains as risk factors for PDD [29–31].

Comparing quantitative MOT parameters among our cognitive groups, we could demonstrate that total error number and processing time were generally higher in PD-CI than in PD-noCI at both visits. This finding is in line with previous studies that state that performance-based tests differentiate between PD patients suffering from different stages of cognitive impairment [6, 10, 11, 13].

Moreover, our data indicates that PD patients worsening in ADL function seems to be primarily characterized by trial and error behavior and skipping a specific part of the chain of action. This behavior might reflect at least partly executive dysfunction, which has been suggested to be a main predictor of ADL impairment [32]. Frequency of omission errors worsened to a greater extent in PD-CI than in PD-noCI over the study period, making the MOT able to differentiate between PD patients with and without cognitive impairment. Therefore, our data support the hypothesis that performance-based ADL tests are sensitive to assess cognitive-driven ADL impairment in PD [11–13].

Since increase in omission errors from baseline to follow-up was associated with new onset PDD, the MOT might help to identify PD-MCI patients who are at potential high risk of having progressed to dementia.

We think that performance-based tests are an important method for evaluation of ADL impairment in PD. We acknowledge PD-specific self-report instruments that are designed to focus on the cognitive ability of the patient, trying to remove the motor demand which is present in most self-report instruments designed for demented patients or Alzheimer`s disease patients [33, 34]. Performance-based tests like the MOT allow for direct assessment and observation and offer more precise ability to detect any impact of motor symptoms during the task performance itself. Shulman and colleagues were able to show a discrepancy between self-report and performance-based measurements [8], making objectiveness another possible advantage. A limitation of this study is the fact that even though the MOT is supposed to be an objective test, it might have a bias because the action has to be rated by an examiner. However, focusing on reliability, we were able to conduct the study with a satisfying IRR. Motor impairment as confounding factor for ADL impairment in PD [35] is a challenging factor, which we tried to account for by correcting it in all group comparisons and predictive models. We also decided not to statistically analyze clumsiness errors since motor impairment seems to be a huge contributor to this error. Correlation analysis between MOT and UPDRS-III change scores only showed a marginal association between the increase of the UPDRS-III score and the increase of total processing time and misuse errors,further supporting our conclusion that cognitive impairment rather than motor impairment is mainly accountable for the change in MOT parameters.

The number of PDD patients with follow-up assessment and new-onset PDD and PD-MCI converters were low. The concept of PD-MCI is heterogeneous: Patients with PD-MCI at baseline might progress to PDD, remain in the PD-MCI group or convert back to being classified as cognitively normal [36, 37]. Therefore, our study needs to be validated in larger cohorts with a higher number of PDD patients and thus most probably more converters over the disease course, so that it is possible to examine the different subgroups (PD-MCI reversible, PD-MCI stable and PDD new-onset) individually.

We conclude that the MOT, especially the frequency of omission errors, is a promising tool to rate PD patients objectively and to help detect PD patients with high-risk for having mild cognitive impairment or dementia.

## Supporting information

S1 Table Cognitive profile for each patient group at baseline. Baseline cognitive profile of PD patients with (PD-CI) and without (PD-noCI) cognitive impairment, as well as with mild cognitive impairment (PD-MCI) and dementia (PDD).
(DOCX)

S2 Table Baseline characteristics including the lost to follow-up cohort. Baseline characteristics of all 131 Parkinson’s disease patients and between group comparison of follow-up (n = 73) and lost to follow-up (n = 58) cohort.
(DOCX)

S3 Table Correlation analysis between MOT change scores and change scores of clinical parameters. Correlation between change scores of MOT parameters change scores of the UPDRS-III and neuropsychological data using theSpearman-rank correlation coefficient (rho).
(DOCX)
