# Supplementary material for: The Multiple Object Test as a performance-based tool to assess the decline of ADL function in Parkinson’s disease
Source: PLoS One. 2018 Aug 1;13(8):e0200990. doi: 10.1371/journal.pone.0200990 (PMC6070239; doi:10.1371/journal.pone.0200990)
Supplement: S1 Table — Baseline cognitive profile of PD patients with (PD-CI) and without (PD-noCI) cognitive impairment, as well as with mild cognitive impairment (PD-MCI) and dementia (PDD). (DOCX) [file pone.0200990.s001.docx]

**S1 Table. Cognitive profile for each patient group at baseline.** Baseline cognitive profile of PD patients with (PD-CI) and without (PD-noCI) cognitive impairment, as well as with mild cognitive impairment (PD-MCI) and dementia (PDD).

|  | PD-noCI | PD-CI | *P* value* | PD-MCI | PDD |
| --- | --- | --- | --- | --- | --- |
| Number, n/% | 39/53.4 | 34/46.6 |  | 26/35.6 | 8/11.0 |
| Global Cognition |  |  |  |  |  |
| MMSE, RW | 29/25-30 | 26.5/20-30 | **.001** | 27/22-30 | 25/20-30 |
| PANDA, RW | 25/17-30 | 18/7-30 | **<.001** | 19.5/8-30 | 10.5/7-19 |
| Executive Function |  |  |  |  |  |
| CERAD: Trail Making Test part B | 62/7-100 | 5/0-99 | **.014** | 36/0-99 | 0/0-84 |
| WMS-R: Digit Span Forward | 67/5-98 | 41/2-95 | .06 | 53/2-95 | 28/15-88 |
| NAI: Figure Test | 65/27-95 | 27/2-88 | **.001** | 40.5/2-88 | 14/3-36 |
| Attention |  |  |  |  |  |
| WMS-R: Digit Span Backward | 53/9-93 | 20/2-78 | **<.001** | 20/2-78 | 20/2-53 |
| CERAD: Trail Making Test part A | 66/2-99 | 9/0-100 | **.001** | 14/0-100 | 4/0-58 |
| Memory |  |  |  |  |  |
| CERAD: Word List Memory | 69/10-98 | 8/0-96 | **<.001** | 9/0-96 | 8/0-50 |
| CERAD: Word List Recall | 58/7-96 | 25.5/1-99 | **.007** | 24/1-99 | 48/2-54 |
| CERAD: Word List Intrusion | 69/2-82 | 17/0-84 | **.023** | 21/1-79 | 7/0-84 |
| CERAD: Discriminability | 76/10-86 | 22.5/0-92 | **<.001** | 22.5/0-86 | 22.5/12-92 |
| Visuo-construction |  |  |  |  |  |
| CERAD: Praxis | 66/0-90 | 13/0-88 | .19 | 29/0-88 | 1.5/0-21 |
| CERAD: Praxis Recall | 58/0-96 | 14.5/0-96 | **.007** | 15.5/0-96 | 8/0-90 |
| VOSP: Object Decision | 47.6/7-100 | 38.1/0-91 | **.002** | 38.1/0-91 | 22.5/7-76 |
| Language & Psychomotor speed |  |  |  |  |  |
| CERAD: Verbal Fluency | 46/7-100 | 38/1-93 | .38 | 40/1-93 | 29.5/2-50 |
| CERAD: Boston Naming Test | 79/8-96 | 34.5/0-95 | **.017** | 44/2-84 | 3/0-95 |

Except for the MMSE and PANDA for which raw values are presented, percentile rank scores of each neuropsychological tests are reported. Logistic regression analysis was applied with cognitive groups (PD-noCI, PD-CI) as dependent and the neuropsychological tests as independent variable with the following covariates: years of disease duration, UPDRS-III, and BDI. If not other indicated, values are given as median/ range. PD-noCI, PD patients without cognitive impairment; PD-CI, PD patients with cognitive impairment; PD-MCI, PD patients with mild cognitive impairment; PDD, Parkinson’s disease dementia; n, Number; %, percentage; RW, raw data; MMSE, Mini-Mental State Exam; PANDA, Parkinson Neuropsychometric Dementia Assessment; CERAD, Consortium to Establish a Registry for Alzheimer’s Disease; WMS-R, Wechsler Memory Scale–Revised Edition; NAI, Nürnberger-Alters-Inventar; VOSP, Visual Object and Space Perception Battery. * Significant *p* values (*P*<.005) are given in bold.
