# Supplementary material for: The Multiple Object Test as a performance-based tool to assess the decline of ADL function in Parkinson’s disease
Source: PLoS One. 2018 Aug 1;13(8):e0200990. doi: 10.1371/journal.pone.0200990 (PMC6070239; doi:10.1371/journal.pone.0200990)
Supplement: S2 Table — Baseline characteristics of all 131 Parkinson’s disease patients and between group comparison of follow-up (n = 73) and lost to follow-up (n = 58) cohort. (DOCX) [file pone.0200990.s002.docx]

**S2 Table. Baseline characteristics including the lost to follow-up cohort.** Baseline characteristics of all 131 Parkinson’s disease patients and between group comparison of follow-up (n=73) and lost to follow-up (n=58) cohort.

| Baseline characteristics | All | Follow-up | Lost to follow-up | *P* value* |
| --- | --- | --- | --- | --- |
| Number, n/% | 131/100 | 73/55.7 | 58/44.3 |  |
| Age in years | 70.6/46-89 | 69.3/46-79 | 71.9/57-89 | **<.001** |
| Male gender, n/% | 113/86.3 | 66/90.4 | 47/81.0 | .12 |
| Years of education | 12/6-21 | 12/6-20 | 12/8-21 | .81 |
| Years of disease duration | 6/1-22 | 6/1-22 | 8/1-19 | .14 |
|  |  |  |  |  |
| LEDD | 620/0-2743 | 560/100-2743 | 710/0-2020 | .11 |
| UPDRS-III | 27/7-55 | 22/7-55 | 31/10-51 | **.004** |
| Hoehn & Yahr stage, n/% |  |  |  | **<.001** |
| 1/1.5 | 15/11.5 | 12/16.4 | 3/5.2 |  |
| 2/2.5 | 70/53.4 | 49/67.1 | 21/36.2 |  |
| 3 | 33/25.2 | 9/12.3 | 24/41.4 |  |
| 4 | 13/9.9 | 3/4.1 | 10/17.2 |  |
| BDI | 9/0-40 | 8/0-34 | 10/0-40 | **.026** |

If not other indicated, values are given as median/range. n, Number; %, percentage; LEDD, Levodopa equivalent daily dose; UPDRS, Unified Parkinson’s Disease Rating Scale; BDI, Beck Depression Inventory. * Significant *p* values (*P*<.005) are given in bold.
