# Supplementary material for: The Multiple Object Test as a performance-based tool to assess the decline of ADL function in Parkinson’s disease
Source: PLoS One. 2018 Aug 1;13(8):e0200990. doi: 10.1371/journal.pone.0200990 (PMC6070239; doi:10.1371/journal.pone.0200990)
Supplement: S3 Table — Correlation between change scores of MOT parameters change scores of the UPDRS-III and neuropsychological data using theSpearman-rank correlation coefficient (rho). (DOCX) [file pone.0200990.s003.docx]

**S3 Table. Correlation analysis between MOT change scores and change scores of clinical parameters.**

|  | total error number | total processing time | perplexity errors | omission errors | mislocation errors | misuse errors | sequence errors |
| --- | --- | --- | --- | --- | --- | --- | --- |
| Motor function |  |  |  |  |  |  |  |
| UPDRS-III | .03 (.83) | .23 (.05) | .01(.93) | .07(.58) | -.04 (.72) | **.23 (.050)** | .01 (.95) |
| Global Cognition |  |  |  |  |  |  |  |
| MMSE, RW | -.18 (.12) | -.21 (.07) | -.21 (.07) | -.06 (.62) | -.07 (.55) | **-.24 (.037**) | .10 (.,41) |
| PANDA, RW | .01 (.97) | -.17 (.16) | -.17 (.16) | .10 (.43) | .15 (.23) | -21 (.09) | .15 (.23) |
| Executive Function |  |  |  |  |  |  |  |
| CERAD: Trail Making Test part B | -.03 (.84) | --03 (.83) | -.10 (.41) | -.01 (.95) | -.03 (.80) | .17 (.18) | -.00 (.98) |
| WMS-R: Digit Span Forward | -.06 (.63) | .07 (.56) | **-.27** (.**020**) | .04 (.72) | .21 (.08) | .01 (.91) | .01 (.92) |
| NAI: Figure Test | .15 (.24) | -.14 (.25) | .06 (.64) | .21 (.08) | .09 (.47) | -.08 (.51) | .05 (.69) |
| Attention |  |  |  |  |  |  |  |
| WMS-R: Digit Span Backward | -.07 (.58) | -.01 (.91) | -.14 (.23) | -.09 (.45) | -.04 (.77) | .14 (.24) | .03 (.79) |
| CERAD: Trail Making Test part A | .03 (.81) | **-. 36 (.002)** | -.09 (.48) | .08 (.52) | .18 (.13) | -.02 (.86) | -.01 (.92) |
| Memory |  |  |  |  |  |  |  |
| CERAD: Word List Memory | .07 (.57) | -.02 (.84) | -.12 (.30) | .07 (.56) | .00 (.99) | -.02 (.85) | .12 (.32) |
| CERAD: Word List Recall | .02 (.85) | -.03 (.83) | -.12 (.33) | .02 (.86) | .03 (.82) | .14 (.23) | .14 (.24) |
| CERAD: Word List Intrusion | .08 (0.50) | .12 (.30) | -.11 (.37) | .13 (.27) | .11 (.37) | .03 (.81) | .17 (.15) |
| CERAD: Discriminability | -.11 (.35) | -.15 (0.21) | -.04 (0.77) | -.05 (.67) | .03 (.80) | -.13 (.26) | -.04 (.72) |
| Visuo-construction |  |  |  |  |  |  |  |
| CERAD: Praxis | .11 (.35) | -.04 (.76) | .10 (.40) | .02 (.90) | .09 (.46) | -.17 (.15) | .09 (.45) |
| CERAD: Praxis Recall | -.04 (.73) | **-.26 (.024)** | .02 (.85) | -.22 (.06) | -.06 (.62) | **-.25** (**.030**) | **.28** (**.018**) |
| VOSP: Object Decision | **-.28** (**.018**) | -.21 (.09) | -.13 (.29) | -.12 (.34) | -.19 (.12) | -.04 (.73) | **-.30** (**.011**) |
| Language & Psychomotor speed |  |  |  |  |  |  |  |
| CERAD: Verbal Fluency | .09 (.44) | -.10 (.41) | -.06 (.62) | .11 (.36) | **.30** (**.011**) | -.11 (.37) | .15 (.21) |
| CERAD: Boston Naming Test | .14 (.25)  14 (.25) | .15 (.21) | .05 (.69) | **.28** (**.018**) | **.28** (**.017**) | .01 (.91) | -.07 (.56) |

Except for the UPDRS-II, MMSE and PANDA for which raw values are presented, percentile rank scores of each neuropsychological tests are reported. Correlation analysis was based on the Spearman-rank correlation coefficient (rho). Values are given as rho; 2-sided p values are added in (). UPDRS-III, Unified Parkinson’s Disease Rating Scale III; RW, raw data; MMSE, Mini-Mental State Exam; PANDA, Parkinson Neuropsychometric Dementia Assessment; CERAD, Consortium to Establish a Registry for Alzheimer’s Disease; WMS-R, Wechsler Memory Scale–Revised Edition; NAI, Nürnberger-Alters-Inventar; VOSP, Visual Object and Space Perception Battery. * Significant *p* values (*P*<.005) are given in bold.
